# Ubiquitin-like protein FAT10 promotes bladder cancer progression by stabilizing survivin

**DOI:** 10.18632/oncotarget.12976

**Published:** 2016-10-28

**Authors:** Dingxiang Dong, Weifan Jiang, Jun Lei, Leifeng Chen, Xiuxia Liu, Jin Ge, Ben Che, Xiaoqing Xi, Jianghua Shao

**Affiliations:** ^1^ Department of General Surgery, Second Affiliated Hospital of Nanchang University, Nanchang, 33000, China; ^2^ Department of Urology Surgery, Second Affiliated Hospital of Nanchang University, Nanchang, 33000, China; ^3^ Jiangxi Province Key Laboratory of Molecular Medicine, Nanchang, 33000, China

**Keywords:** bladder carcinoma, FAT10 protein, tumor promoter, stabilize, survivin protein

## Abstract

Human HLA-F adjacent transcript 10 (FAT10) is a member of the ubiquitin-like-modifier family of proteins, which have been implicated in cancer development. In addition, the Survivin protein promotes proliferation in bladder cancer (BC). In this study, we explored the link between FAT10 and Survivin. FAT10 expression was dramatically up-regulated in BC tissue samples, and Kaplan-Meier survival analysis revealed that BC patients with high FAT10 expression had shorter overall survival than those with low FAT10 expression. Moreover, RNAi-mediated FAT10 knockdown decreased Survivin protein levels and inhibited BC proliferation both *in vitro* and *in vivo*. FAT10 directly bound to and stabilized Survivin protein, thereby promoting cancer cell proliferation by inhibiting ubiquitin-mediated degradation. These results reveal a novel mechanism by which FAT10 promotes tumor proliferation by directly stabilizing Survivin protein in BC.

## INTRODUCTION

Bladder cancer (BC) is the most common and most fatal type of urinary tumor [1, 2]. Although 5-year survival rates in BC patients have improved, one third of all patients still experience recurrence. Emerging evidence suggests that BC recurrence is related to cell proliferation [3, 4], and the molecular mechanisms that regulate proliferation in BC are a topic of current research.

HLA-F locus adjacent transcript 10 (FAT10), a member of the ubiquitin-like protein (UBL) family, contains 165 amino acids consisting of two in-tandem ubiquitin-like domains [5]. FAT10 is involved in various essential cellular development processes, including immune-mediated inflammation, apoptosis, cell cycle progression, and proliferation [6–8]. In recent years, studies have shown that FAT10 also promotes tumor development and proliferation [9]. FAT10 expression is elevated in liver cancer, stomach cancer, glioma, and other cancer tissues [6, 10, 11]. We recently demonstrated that FAT10 protein is expressed in many tumor cells, and high FAT10 levels were associated with increased proliferation in hepatocellular carcinoma, colon cancer, and cervical carcinoma cells [12, 13]. However, the role of FAT10 expression in BC cells remains unclear.

Survivin, a member of the inhibitor of apoptosis protein (IAP) family, also plays an important role in human cell growth, apoptosis, and death [14, 15]. In addition, the ubiquitin proteasome pathway degrades Survivin protein [16]. Survivin is overexpressed in colon cancer, liver cancer, lung cancer, cervical carcinoma, and BC [14, 17–20]. Overexpression of Survivin is associated with increases in tumor stage and grade and may promote cell proliferation and serve as a predictive marker of overall survival (OS) in BC [17]. However, the mechanism by which Survivin expression is regulated in BC remains unclear.

In this study, we found that FAT10 expression was up-regulated in BC tissues, and increased FAT10 expression was associated with poor prognosis. Additionally, FAT10 facilitated BC proliferation by up-regulating Survivin protein levels and directly stabilizing Survivin protein.

## RESULTS

### FAT10 expression is up-regulated in BC tissues and is associated with BC progression

**Figure 1:**
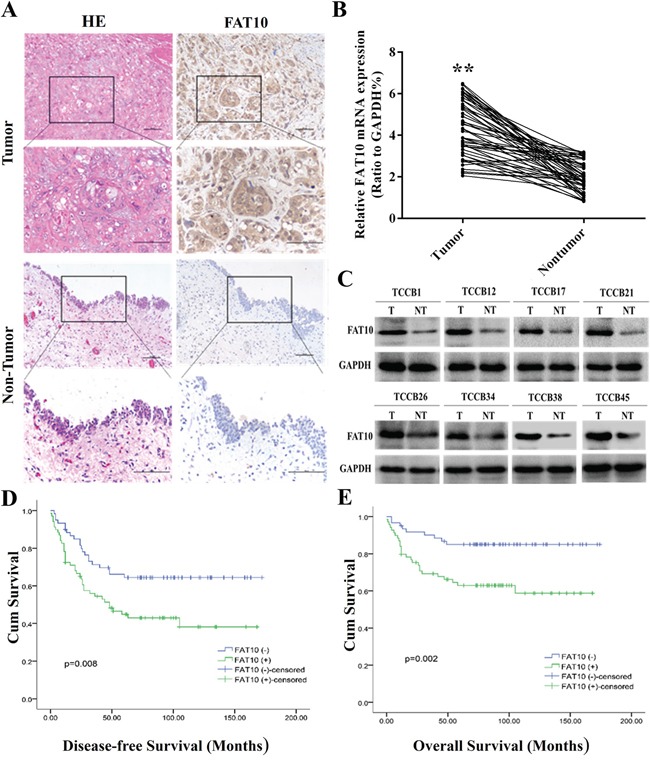
High FAT10 expression is associated with poor prognosis in bladder cancer patients **A.** IHC showed that FAT10 protein levels were increased in BC tissues (magnification: 100×, inset magnification: 400×). **B** and **C.** qRT-PCR and western blot analysis of FAT10 mRNA and protein levels in BC tissue samples and corresponding adjacent tissues. **D** and **E.** Kaplan-Meier survival curves for 133 BC patients. Curves are shown for patients with FAT10-positive tumors and FAT10-negative/weak tumors (**P < 0.01).

We first examined FAT10 expression in 133 BC tissue samples and corresponding adjacent tissues using immunohistochemistry (IHC). FAT10 protein was highly expressed in 54.14% (72/133) of the BC tissue samples (Figure 1A). We then examined FAT10 expression in 50 BC tissue samples and corresponding adjacent normal tissues using qRT-PCR and western blotting. qRT-PCR revealed that FAT10 mRNA expression was increased in BC tissues compared to corresponding adjacent tissues (Figure 1B), and western blots showed that FAT10 protein was overexpressed in 68% (34/50) of the BC tissue samples (Figure 1C). These results indicate that FAT10 mRNA and protein levels are upregulated in BC tissues.

Next, we analyzed associations between FAT10 protein overexpression and clinicopathological parameters in BC patients. FAT10 overexpression was associated with age and increases in tumor size, number, and stage (Table 1). Furthermore, Kaplan-Meier survival analysis revealed that patients with higher FAT10 expression in BC tissues had poorer survival than those with lower FAT10 expression (Figure 1D and 1E). Taken together, these results indicate that FAT10 is aberrantly up-regulated in BC tissues, and this upregulation is associated with increases in BC progression.

**Table 1:** FAT10 expression in 133 bladder cancer patients

Patients (n=133)		FAT 10		
		-	+	P value
Gender				0.653
Female	24	12(50.0%)	12(50.0%)	
Male	109	49(44.9%)	60(55.1%)	
Age (years)				0.034
0-59	54	31(57.4%)	23(42.6%)	
≥60	79	30(38.0%)	49(62.0%)	
Tumor size(cm)				0.033
<3	80	43(53.8%)	37(46.2%)	
≥3	53	18(33.9%)	35(66.1%)	
Tumor number				0.031
<3	82	44(53.7%)	38(46.3%)	
≥3	51	17(33.3%)	34(66.7%)	
Grade				0.113
G1	56	29(51.7%)	27(48.3%)	
G2-G3	77	32(41.6%)	45(58.4%)	
Tumor stage				0.016
Ta-T1	90	48(53.3%)	42(46.7%)	
T2-T4	43	13(30.2%)	30(69.8%)	

### FAT10 promotes human BC cell proliferation *in vitro* and *in vivo*

**Figure 2:**
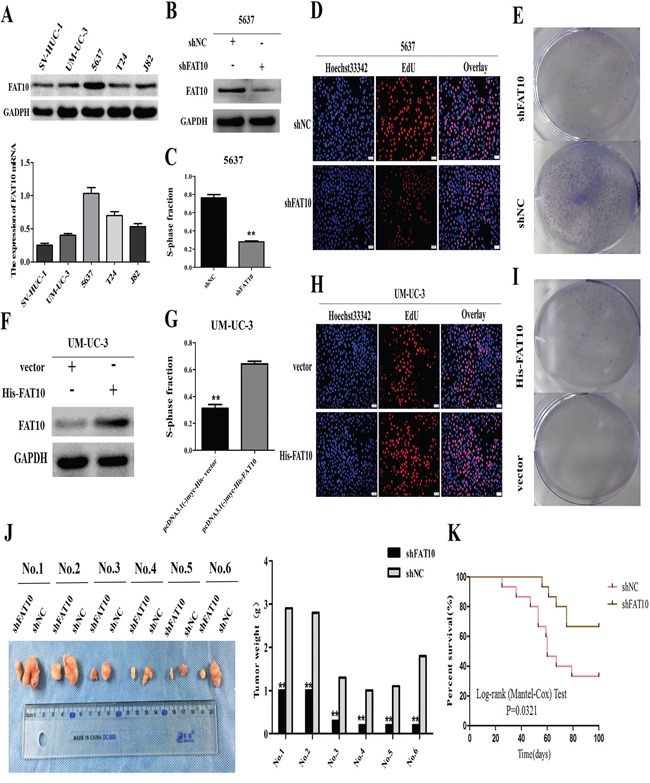
FAT10 promotes BC proliferation *in vitro* and *in vivo* **A.** Western blot and RT-qPCR analysis of FAT10 expression in bladder cell lines. **B** and **F.** Western blot analysis of FAT10 expression in 5637 cells transfected with the shFAT10 plasmid (B) or the His-FAT10 plasmid (F). **C, D, E, G, H,** and **I.** BC cell proliferation was measured using EdU and clone formation assays in 5637 cells transfected with the shFAT10 plasmid (C, D, and E) or the His-FAT10 plasmid (G, H, and I). (scale bar, 50μm). **J** and **K.** Stable FAT10 knockdown inhibited the tumorigenicity of 5637 cells in nude mice (means ± SEM, n=6, **P<0.01).

To further explore the role of FAT10 in BC, we examined FAT10 levels in various BC cell lines using western blotting and qRT-PCR analysis. FAT10 expression was higher in BC cells (UM-UC-3, 5637, T24 and J82) than in normal bladder epithelial cells (SV-HUC-1) (Figure 2A). Next, to determine whether FAT10 was involved in BC cell proliferation, we stably transfected a FAT10-specific short hairpin RNA (shFAT10) into 5637 cells. Western blot analysis revealed that downregulation of FAT10 reduced FAT10 protein levels in these cells (Figure 2B). Edu and colony formation assays confirmed that downregulation of FAT10 inhibited proliferation in 5637 cells compared to the NC group (Figure 2C-2E). Conversely, when UM-UC-3 cells were transfected with the FAT10 plasmid, cell proliferation increased as FAT10 expression increased (Figure 2F-2I). These results indicate that FAT10 promotes BC cell proliferation *in vitro*.

Finally, we examined the effects of FAT10 on BC *in vivo* by establishing a nude mouse tumorigenicity assay. Tumors formed after injection of 5637-shFAT10 cells were smaller and weighed less compared to tumors formed after injection of control cells (Figure 2J). Furthermore, mice that received 5637-shFAT10 cell injections survived longer than control group mice (Figure 2K). Taken together, these data indicate that FAT10 promotes human BC cell proliferation both *in vitro* and *in vivo.*

### FAT10 promotes BC cell proliferation by increasing survivin expression

**Figure 3:**
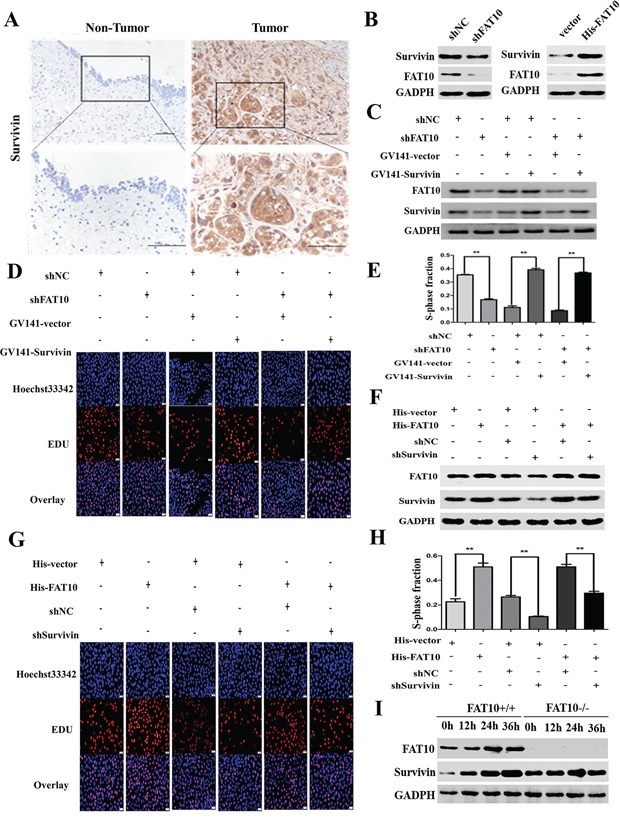
FAT10 promotes Survivin expression and proliferation in BC cells **A.** IHC showed that Survivin protein was upregulated in BC tissue (magnification: 100×, inset magnification: 400×). **B.** Western blot analysis of Survivin expression in BC cell lines transfected with the shFAT10 plasmid or the His-FAT10 plasmid. **C.** Survivin overexpression attenuated the reduction in Survivin expression in 5637-shFAT10 cells. **D** and **E.** Edu assays showed that Survivin overexpression rescued cell proliferation in 5637-shFAT10 cells. (**P<0.01). **F.** Survivin knockdown successfully decreased Survivin expression in UM-UC-3-FAT10 cells. **G** and **H.** Edu assays showed that Survivin knockdown decreased proliferation in UM-UC-3-FAT10 cells (**P<0.01). **I.** Survivin levels were detected in IFN-γ/TNF-α-treated FAT10 KO cells by western blotting.

Studies have demonstrated that Survivin plays an important role in BC proliferation; we therefore speculated that FAT10 might affect BC proliferation by regulating the expression of Survivin. The expression of both FAT10 and Survivin was significantly elevated in BC tissues (Figure 1A and 3A), and scatter plots showed that FAT10 and Survivin expression levels were positively correlated in BC tissues (Supplementary Figure 1A-1C). To investigate whether FAT10 regulates Survivin expression in BC cells, we stably transfected the shFAT10 plasmid into 5637 cells. While FAT10 downregulation reduced Survivin protein levels in 5637 cells (Figure 3B), Survivin mRNA expression was not affected by FAT10 downregulation (Supplementary Figure 1D).

To confirm that FAT10 mediates BC proliferation by regulating Survivin, we increased Survivin expression in FAT10 knockdown cancer cells and analyzed FAT10 and Survivin protein levels and cell proliferation using western blotting and Edu assays. As illustrated in Figure 3C, the results showed that the down-regulation of FAT10 decreased Survivin expression, whereas the up-regulation of Survivin attenuated the loss of Survivin expression in FAT10 knockdown 5637 cells. We also found that the knockdown of FAT10 dramatically decreased the proliferation abilities of 5637, whereas the up-regulation of Survivin rescued the decreased proliferation abilities induced by FAT10 knockdown (Figure 3D and 3E).

Next, we decreased Survivin expression in FAT10-overexpressing BC cells and measured FAT10 and Survivin protein levels and proliferation. Overexpression of FAT10 increased Survivin levels, while Survivin knockdown dramatically inhibited the FAT10-induced increase in Survivin expression in UC-UM-3 cells. Meanwhile, the down-regulation of Survivin reduced FAT10-induced increases in cell proliferation (Figure 3F-3H). Finally, we used FAT10-KO HEK293 cells to further demonstrate this phenomenon. When FAT10-KO HEK293T cells were treated with IFN-γ/TNF-α, the expression of Survivin was unchanged (Figure 3I). These results demonstrate that Survivin is required for FAT10-mediated BC cell proliferation.

### FAT10 stabilizes survivin expression by inhibiting ubiquitin-mediated degradation

**Figure 4:**
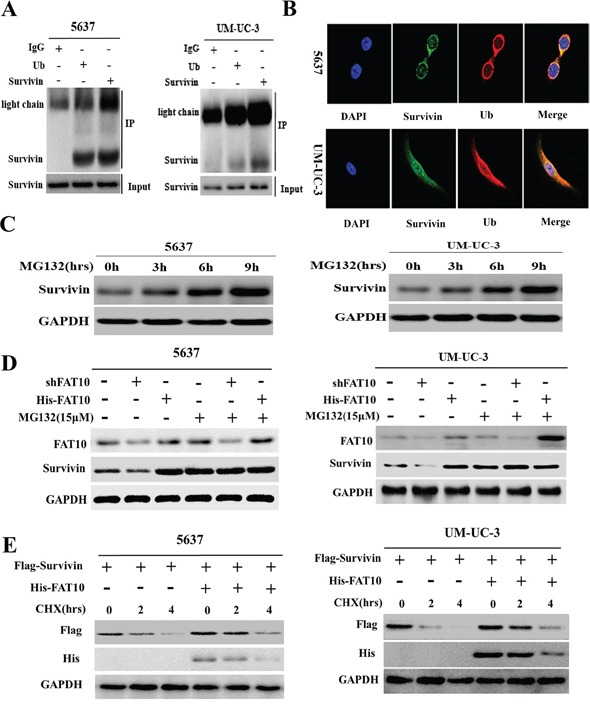
FAT10 increases Survivin levels by inhibiting Survivin protein degradation in BC cells **A.** Co-IP for Survivin and ubiquitin in 5637 and UM-UC-3 cells. **B.** Co-localization of ubiquitin and Survivin in 5637 and UM-UC-3 cells; ubiquitin (1:200) in red, Survivin (1:200) in green, DAPI nuclear counterstaining in blue. **C** and **D.** 5637 and UM-UC-3 cells were treated with MG132 (15 μM) for the indicated times, and Survivin levels were detected by western blotting. **E.** 5637 and UM-UC-3 cells were treated with CHX (20 μM) for the indicated times, and Survivin levels were detected by western blotting.

Although FAT10 increased Survivin protein levels, it did not affect Survivin mRNA expression. Previous research suggests that Survivin is degraded by the ubiquitin-proteasome system (UPS) [16]. Thus, we speculated that FAT10 might regulate Survivin expression by inhibiting ubiquitin-mediated Survivin degradation. We first investigated the interaction between endogenous Survivin and ubiquitin in BC cells using Co-IP and confocal microscopy. Endogenous Survivin and ubiquitin bound directly to each other in 5637 and UM-UC-3 cells (Figure 4A and 4B). Furthermore, cumulative Survivin levels increased as the duration of treatment with MG132, an inhibitor of proteasome pathway-dependent protein degradation, increased in both 5637 and UM-UC-3 cells (Figure 4C). These results demonstrate that degradation of Survivin protein in BC cells is mediated by the UPS.

Next, to determine whether FAT10 affects Survivin protein degradation in BC, we transfected the shFAT10 and His-FAT10 vectors into 5637 and UM-UC-3 cells treated with or without MG132 and measured Survivin expression. Altering FAT10 expression had no effect on Survivin expression in 5637 and UM-UC-3 cells treated with MG132 (Figure 4D), suggesting that FAT10 does not influence Survivin protein synthesis. To determine whether FAT10 is involved in Survivin degradation, 5637 and UM-UC-3 cells transfected with Flag-Survivin and His-FAT10 plasmids were exposed to CHX, and exogenous Flag-Survivin protein was measured after 0, 2, and 4 h. A degradation dynamics assay revealed that transfection with exogenous His-FAT10 plasmid enhanced FAT10 expression and clearly slowed the rate of Flag-Survivin protein degradation (Figure 4E). These results demonstrate that FAT10 stabilizes Survivin expression by inhibiting ubiquitin-mediated degradation.

### FAT10 decreases survivin ubiquitination by competing with ubiquitin for binding

**Figure 5:**
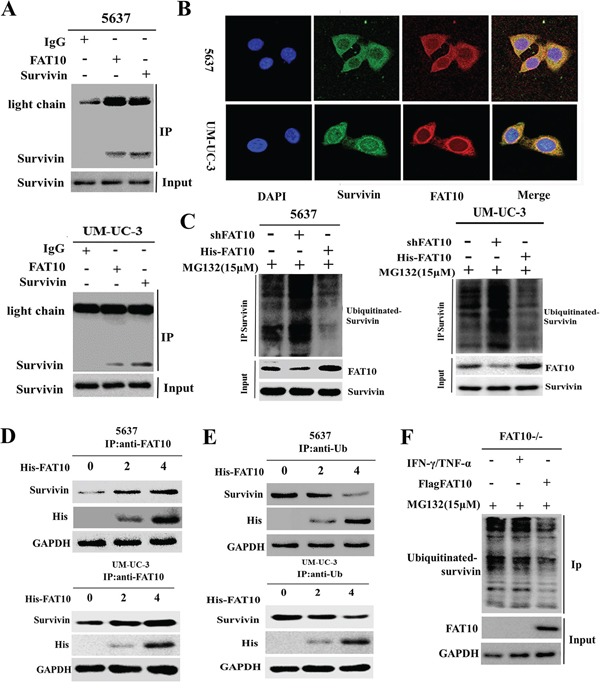
FAT10 stabilizes Survivin by competing with ubiquitin for binding **A.** Co-IP for Survivin and FAT10 in 5637 and UM-UC-3 cells. **B.** Co-localization of FAT10 and Survivin in 5637 and UM-UC-3 cells; FAT10 (1:200) in red, Survivin (1:200) in green, DAPI nuclear counterstaining in blue. **C.** 5637 and UM-UC-3 cells were transfected with the shFAT10 plasmid or the His-FAT10 plasmid and then treated with MG132. Survivin ubiquitination was then measured. **D** and **E.** 5637 and UM-UC-3 cells were transfected with increasing amounts of the His-FAT10 plasmid. Cell lysates were used for Co-IP with anti-Ub or anti-FAT10 beads to detect Survivin binding. **F.** FAT10 KO cells were treated with IFN-γ/TNF-α or transfected with the His-FAT10 plasmid and then treated with MG132. Survivin ubiquitination was then measured.

We previously found that FAT10 stabilizes substrates by competing with ubiquitin (Ub) for binding, ultimately decreasing substrate ubiquitination [12, 13]. Thus, we hypothesized that competition between FAT10 and Ub for Survivin binding decreases the ubiquitination and degradation of Survivin in BC cells. To test this hypothesis, we first determined whether the FAT10 and Survivin proteins interacted directly. Co-IP and confocal microscopy revealed a direct interaction between the FAT10 and Survivin proteins (Figure 5A and 5B). To assess the role of FAT10 in Survivin degradation, we then treated 5637 and UM-UC-3 cells with MG132 to inhibit proteasome-mediated protein degradation and then incubated cell lysates with a Survivin antibody. Co-IP results revealed that the knockdown of FAT10 resulted in an increase in the ubiquitination level of endogenous Survivin and that the overexpression of FAT10 led to a decrease in the ubiquitination level of endogenous Survivin (Figure 5C). These results demonstrate that FAT10 stabilizes Survivin expression by inhibiting Survivin ubiquitination in BC.

To explore the mechanism by which FAT10 stabilizes Survivin expression and inhibits Survivin ubiquitination, 5637 and UM-UC-3 cells were transfected with increasing amounts of His-FAT10 plasmid. Cell lysates were then used for immunoprecipitation with anti-Ub and anti-FAT10 beads to detect Survivin binding. As FAT10 protein levels increased, FAT10-Survivin complex levels gradually increased, while Ub-Survivin complexes levels gradually decreased. Conversely, as Ub levels increased, FAT10-Survivin complex levels gradually decreased, while Ub-Survivin complex levels gradually increased (Figure 5D and 5E). These experiments demonstrate that FAT10 and Ub compete for binding with Survivin. Finally, in FAT10-KO cells, Co-IP results showed that IFNγ/TNFα treatment did not change Survivin ubiquitination levels. However, when FAT10 expression was restored in FAT10-KO cells using His-FAT10, Survivin ubiquitination decreased (Figure 5F). These results confirm that FAT10 overexpression decreases the formation of Ub-Survivin complexes, in turn increasing Survivin expression.

## DISCUSSION

FAT10, a member of the UBL family, is involved in cell cycle regulation, apoptosis, and aberrant mitosis [22–25]. Recent evidence suggests that abnormally high FAT10 expression is common cancer, and FAT10 may serve as a novel cancer biomarker. For example, Liu *et al*. found that FAT10 is an independent risk factor in hepatocellular carcinoma [26]. Additionally, Sub *et al*. found that FAT10 overexpression promotes tumor progression, and FAT10 may be a novel biomarker in pancreatic ductal adenocarcinoma (PDAC) [27]. However, the expression of FAT10 in BC and its effects on patient survival remain unknown. In this study, we found that FAT10 expression was significantly upregulated in BC tissues, and FAT10 overexpression was associated with poor prognoses in BC patients. Furthermore, FAT10 promoted BC cell proliferation both *in vitro* and *in vivo*. These results suggest that FAT10 may promote tumorigenesis and serve as a novel biomarker in BC.

Next, we investigated the mechanism by which FAT10 affects proliferation in BC cells. Survivin, which is highly expressed and promotes tumor proliferation in BC, is a highly specific biomarker used to detect BC [27]. Ye *et al*. reported that ERK and AKT signaling co-regulate the transcription of Survivin during metastatic progression in colorectal cancer [30]. Another study found that ALKBH8 promotes BC growth and progression by increasing Survivin expression [31]. In this study, we identified a novel mechanism by which FAT10 promotes BC cell proliferation by upregulating Survivin expression. First, FAT10 and Survivin expression were positively correlated with each other and were both elevated in BC tissues. Moreover, FAT10 knockdown reduced Survivin expression and decreased proliferation, and increasing Survivin expression restored the proliferative capacity of FAT10-knockdown BC cells. In addition, downregulation of Survivin expression inhibited the FAT10 overexpression-induced increase in BC proliferation.

Finally, we investigated the mechanism by which FAT10 regulates Survivin levels. FAT10 belongs to a class of UBLs that have a three-dimensional core structure similar to that of ubiquitin (Ub) [32], and studies of FAT10 have focused on protein degradation [33]. Recently, we showed for the first time that FAT10 stabilized substrate expression by inhibiting ubiquitination of the substrate [13]. In that study, we demonstrated that FAT10 and Ub competitively bound to the substrate to form FAT10- and Ub-substrate complexes; FAT10 overexpression decreased Ub-substrate complex levels and increased FAT10-substrate complex levels [12]. In this study, we provide evidence that FAT10 also stabilizes Survivin and thus increases Survivin expression in BC cells. First, while FAT10 did not influence Survivin protein synthesis, it did inhibit ubiquitin-mediated degradation of Survivin. Second, the levels of ubiquitin-conjugated Survivin were significantly decreased in the presence of FAT10 overexpression. In contrast, the levels of ubiquitin-conjugated Survivin were significantly increased in the presence of FAT10 downregulation. Third, high FAT10 expression results in reduced levels of Ub-Survivin conjugates that decrease the Survivin ubiquitination level, resulting in decreased ubiquitination-mediated Survivin degradation.

In conclusion, we identified a novel mechanism by which FAT10 promotes tumor proliferation by stabilizing Survivin expression in BC. These findings suggest that novel therapeutic strategies targeting FAT10 may be beneficial in the treatment of BC.

## MATERIALS AND METHODS

### Cell culture, plasmids, and reagents

The SV-HUC-1 and human cancer T24, J82, 5637, and UM-UC-3 cell lines were purchased from the Shanghai Cell Bank, Type Culture Collection Committee of Chinese Academy of Science (Shanghai, China). All of these cell lines were authenticated by the Cell Bank using short tandem-repeat profiling. Only cells that had been received within six months were used in the study. Cells were cultured in Dulbecco's modified Eagle's medium (DMEM) (Gibco Laboratories, Grand Island, NY, USA) supplemented with fetal bovine serum (FBS) (Gibco Laboratories, Grand Island, NY, USA) and were exposed to antibiotics at 37°C with 5% CO_2_. The primers are provided in Supplementary Table 1. The identification of the interference effects are included in Supplementary Figure 2

### Patients and tissue specimens

BC specimens were collected from 133 patients who underwent BC resection at the Second Affiliated Hospital of Nanchang University between January 2001 and December 2009. Informed consent was obtained from each patient, and the study protocol was approved by the Ethics Committee of the Second Affiliated Hospital of Nanchang University.

### Immunohistochemistry

The BC and adjacent tissues were fixed, embedded, sectioned, and deparaffinized. Some of the deparaffinized sections were stained with H&E. FAT10 and Survivin detection was performed using paraffin sections and anti-FAT10 and anti-Survivin polyclonal antibodies (Abcam, 1:250 dilution). A peroxidase/3,3'-diaminobenzidine-conjugated secondary antibody was used according to the manufacturer's instructions for visualization. FAT10 and Survivin levels were subjectively graded based on relative nuclear staining intensity.

### Real-time quantitative polymerase chain reaction (qRT-PCR), western blot analysis, immunofluorescence, and co-immunoprecipitation (Co-IP)

qRT-PCR, western blotting, immunofluorescence, and Co-IP were performed as previously described [34].

### Generation of CRISPR-Cas9 knockout cell lines

To design specific target sequences for shRNA synthesis, the following primers were used: FAT10#1 forward (5' to 3') CACCgcatgtccgttccgaggaat; FAT10#1 reverse (5' to 1663') AAACattcctcggaacggacatgc; FAT10#2 forward (5' to 3') CACCgcaatgatcgagactaagac; FAT10#2 reverse (5' to 3') AAACgtcttagtctcgatcattgc. FAT10 knockout cell lines were generated as previously described [12].

### Edu assay

BC cells were incubated with 5-ethynyl-20-deoxyuridine (EdU) (Ribobio, Guangzhou, China) for 5 hours and were subsequently processed according to the manufacturer's instructions. Edu assays were performed as previously described [12, 35].

### Tumorigenicity assay

5637 cells (5×10^6^ cells) stably transfected with shFAT10 or empty vector were subcutaneously injected into the flanks of BALB/c nude male mice (Hunan SJA Laboratory Animal Co., Ltd.). Tumor weights are presented as means ± SD. The animal work was approved by the Ethics Committee for Animal Experiments of the Second Affiliated Hospital of Nanchang University.

### Statistical analysis

All data were analyzed using SPSS (Statistical Package for the Social Sciences) 19.0 (SPSS, Inc.). The patient survival curve was calculated using the Kaplan-Meier method. Differences between groups were analyzed using Student's *t*-tests when comparing two groups or one-way analyses of variance (ANOVA) when comparing more than two groups. Differences were considered statistically significant at *P* < 0.05.

## SUPPLEMENTARY MATERIALS FIGURES AND TABLE



## Abbreviations

FAT10: Human HLA-F adjacent transcript 10
BC: bladder cancer
UBLs: ubiquitin-like protein family
IAP: inhibitor of apoptosis protein
IHC: immunohistochemistry
RT-PCR: reverse transcription polymerase chain reaction
qRT-PCR: quantitative RT-PCR
KO: knockout
PDAC: pancreatic ductal adenocarcinoma.
