# Supplementary material for: Ubiquitin-like protein FAT10 promotes bladder cancer progression by stabilizing survivin
Source: Oncotarget. 2016 Oct 28;7(49):81463–73. doi: 10.18632/oncotarget.12976 (PMC5348406; doi:10.18632/oncotarget.12976)
Supplement: Supplementary file 1 [file oncotarget-07-81463-s001.pdf]

Ubiquitin-like protein FAT10 promotes bladder cancer progression by stabilizing survivin

SUPPLEMENTARY FIGURES AND TABLE

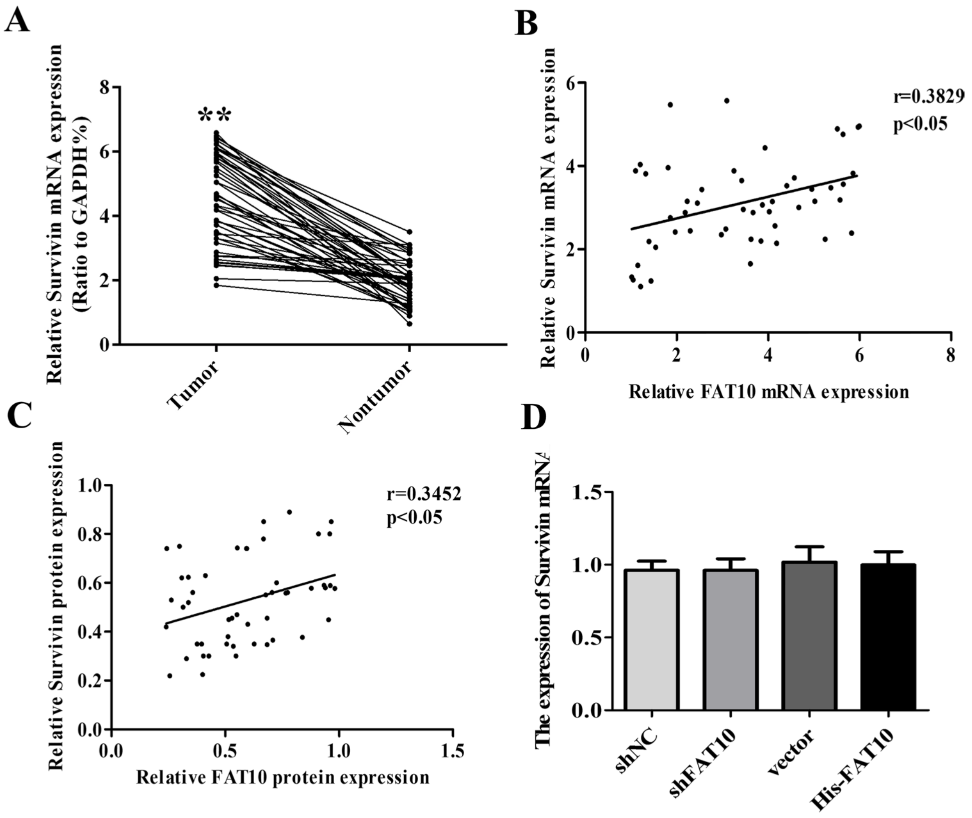

Supplementary Figure S1: A positive correlation between FAT10 and Survivin in bladder cancer patients: Survivin mRNA expression in bladder cancer cell lines transfected with the shFAT10 plasmid or His-FAT10 plasmid. A. The expression of Survivin mRNA were detected in bladder cancer tissue samples and the corresponding adjacent tissues by qRT-PCR. **B** and **C.** Scatter plots show a positive correlation between FAT10 and Survivin at the mRNA and protein level in 133 bladder cancer patients (r=0.3829, *P*<0.001 and r=0.3452, *P*<0.001). **D.** qRT-PCR analysis of Survivin mRNA expression in bladder cancer cell lines transfected with the shFAT10 plasmid or His-FAT10 plasmid.

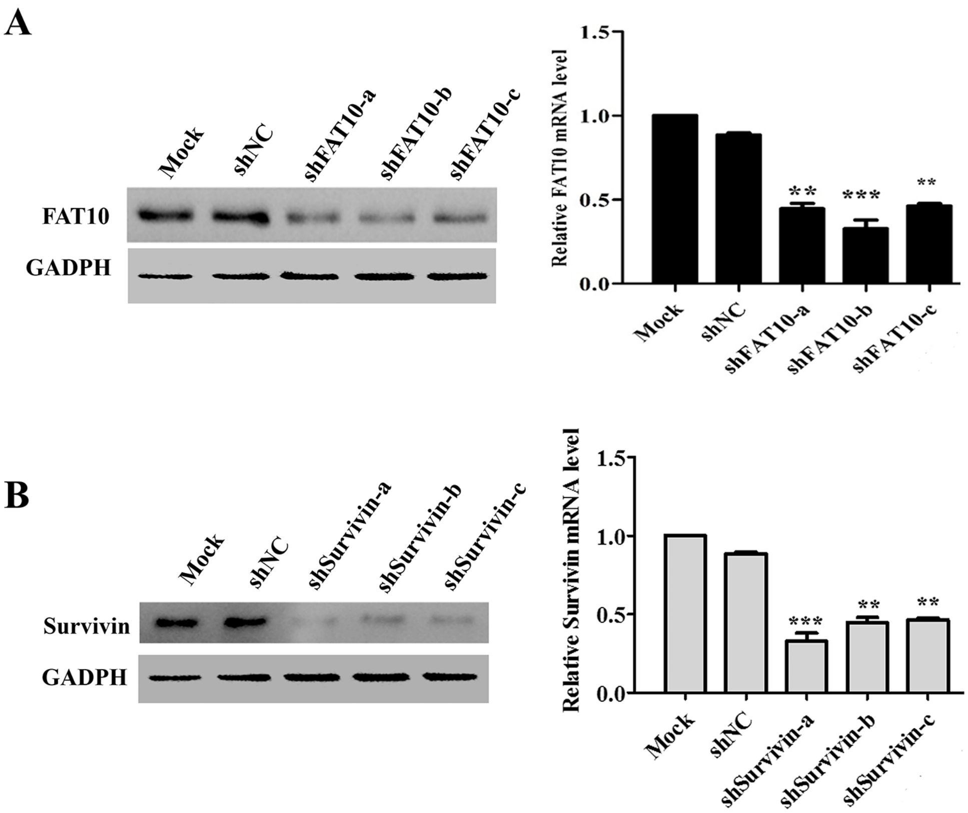

Supplementary Figure S2: shRNA plasmids. **A** and **B.** FAT10, Survivin, expression decreased after transfection of the corresponding shRNA plasmids.

Supplementary Table S1: Primers and shRNA target sequences

Name	sequences	Enzyme
**Primers for real-time PCR:**		
FAT10 sense	5'-CTTGTGGAGTCAGGTGATG-3'	
FAT10 antisense	5'-CCATTGCAAGTCACAATCTG-3'	
Survivin sense	5'-TGACGACCCCATAGAGGAACA-3'	
Survivin antisense	5'- CGCACTTTCTCCGCAGTTTC-3'	
GAPDH sense	5'-CTTCATTGACCTCAACTACA-3'	
GAPDH antisense	5'-ACTCCACGACATACTCAGC-3'	
**Primers for plasmids construct:**		
pcDNA3.1(-)myc-His-FAT10 sense	5'-GGAATTCGCCACCATGGCTCCCAATGCTTCCTGC-3'	EcoRI
pcDNA3.1(-)myc-His-FAT10 antisense	5'-CCCAAGCTTCCCTCCAATACAATAACATGC-3'	Hind III
GV141-Survivin	5'-ACGGGCCCTCTAGACTCGAGCGCCACCATGGGTGCC CCGACGTTGCC-3'	XhoI
GV141-Survivin	5'-AGTCCAGTGTGGTGGAATTCATCCATGGCAGC CAGCTGCTCG-3'	EcoRI
**The target sites of shRNA:**		
shFAT10-a	GGCAGATTACGGCATCAGA	
shFAT10-b	GGGATTTAATGACCTTTGA	
shFAT10-c	GGAGAAGCCTCTCATCTTA	
shSurvivin-a	GCATCTCTACATTCAAGAA	
shSurvivin-b	CCAACAATAAGAAGAAAGA	
shSurvivin-c	CCACTGAGAACGAGCCAGA	
shNC	TTCTCCGAACGTGTCACGT	
shNC	TTCTCCGAACGTGTCACGT	
